# Family-Centered Treatment Program for Problematic Gaming and Excessive Screen Use in a Clinical Child and Youth Population (FAME): Protocol for a Feasibility Pilot Mixed Method Study

**DOI:** 10.2196/56387

**Published:** 2024-10-08

**Authors:** Marie Werner, Sabina Kapetanovic, Emma Claesdotter-Knutsson

**Affiliations:** 1 Department of Clinical Sciences Lund Faculty of Medicine Lund University Lund Sweden; 2 Department of Child and Adolescent Psychiatry Skåne University Hospital Lund Sweden; 3 Department of Behavioral Studies University West Trollhättan Sweden; 4 Department of Psychology Stockholm University Stockholm Sweden

**Keywords:** gaming, family program, family intervention, pilot study,, adolescent, problematic gaming, excessive screen time, children, screen use, child-parent relationship, motivational interviewing

## Abstract

**Background:**

Screen time among children and adolescents has increased dramatically, raising concerns about its impact on development and mental health. While research highlights both potential benefits and risks, excessive use has been linked to issues like anxiety, depression, and gaming addiction. Despite growing concern, effective interventions are scarce. Recognizing the importance of family dynamics in child development, we propose a family-centered program to address problematic gaming and excessive screen use in a clinical population. By involving both children and parents, we aim to create a more comprehensive approach to prevention and treatment.

**Objective:**

This study aims to determine the possibility of distributing and evaluating a family-centered group program for problematic gaming and excessive screen use (FAME) in a clinical child and adolescent psychiatry (CAP) population. We will monitor the recruitment rate; track the retention and attendance rates of both parents and children; and assess whether each session’s objectives are met, the content is delivered within the allotted time, and the necessary resources (eg, facilitators and materials) are available. Additionally, we will gather qualitative and quantitative feedback from participants through postprogram surveys and individual interviews with both children and parents.

**Methods:**

A total of 10 families with ongoing contact with CAP in Skåne, Sweden, will be recruited and offered participation in a family-centered group program targeting children aged 10-18 years with reported difficulties regarding screen gaming or screen use. The intervention to be tested is a newly developed, family-centered, psychoeducational, cognitive behavioral therapy–based intervention addressing both positive and negative aspects of screen use; setting boundaries; the connection between thoughts, feelings, and behaviors; conflict triggers; and sleep hygiene. The primary goal of the pilot study is to test the feasibility of the program, as well as recruitment and the analysis of participants’ experiences with the program.

**Results:**

A total of 11 children and their parents were enrolled during first quarter of 2024. A 4-session pilot was delivered in first quarter of 2024, and the first results are expected in the third quarter of 2024.

**Conclusions:**

The overarching goal of this pilot study is to determine the possibility of distributing and evaluating a family-centered group program for problematic gaming and excessive screen use (FAME) in a clinical CAP population. The insights gained from this study will guide our future research, which will focus on conducting a larger-scale evaluation of the intervention’s impact on family screen time conflicts and inform future strategies for the implementation of family-centered interventions in child and youth clinics.

**Trial Registration:**

ClinicalTrials.gov NCT06098807; https://clinicaltrials.gov/study/NCT06098807

**International Registered Report Identifier (IRRID):**

DERR1-10.2196/56387

## Introduction

During the past 10 years, the use of screens, as in social media and video games, has become one of the most common leisure activities for children and adolescents. According to the Swedish Internet Foundation, 94% of children in Sweden between ages 8 and 12 use screens every day [1], with boys playing video games while girls being more active on social media [2]. The growing trend of children and youth spending excessive amounts of time engaged with screens has been perplexing adults. Parents and professionals who work closely with children and youth are particularly concerned about the potential negative impacts of screen time on their overall development [3-5]. Some research suggests that screen time in general and gaming in particular is an educational and skills training activity that can stimulate the user’s cognitive abilities and ability to solve problems [6]. Other research has concerns that screen use can become a potentially pathological activity that interferes with everyday life with risks of developing psychiatric disorders such as social media disorder or internet gaming disorder (IGD) [7]. Indeed, while there is currently no official diagnosis for social media disorder, IGD is included in the *Diagnostic and Statistical Manual of Mental Disorders, Fifth Edition*, as a condition necessitating further clinical experience and research before inclusion as a formal disorder. The World Health Organization, on the other hand, has included gaming disorder in the *International Classification of Diseases, 11th Revision*, as a formal diagnosis [8].

Screen time for adolescents is a complex issue. While it is often associated with gaming, it encompasses a wider range of activities like social media, communication, and even creative pursuits through apps. Social media use, for instance, can foster positive digital behavior and a sense of belonging [9,10]. However, research also indicates that excessive screen time can negatively impact mental health, leading to anxiety and depression in young people [11]. The increasing presence of social media in teenagers’ lives presents a double-edged sword. Moderate use can be beneficial for social-emotional development and peer relationships. However, problematic social media use, characterized by addictive tendencies and an inability to regulate use, has been consistently linked to depression, anxiety, and stress [12,13]. While not a formal diagnosis, it is often evaluated based on such addictive symptoms [14].

There is a general consensus among school health professionals, social workers, child and adolescent psychiatrists, and even parents that excessive screen time can impact childhood development [15]. However, clear recommendations remain elusive. Many parents allow their children’s current behavior, believing that media content can be educational and that screen time offers a readily available, affordable form of entertainment [16].

Experiencing addictive problems as a child may pose a threat to future psychosocial development [17], including mental health and socioemotional development. In that sense, child and youth mental health services need to put more effort into developing interventions that target children at risk to prevent the development of mental health problems among children and youth. To date, there are no evaluated preventive programs for children and youth with excessive screen time, including problematic gaming, in Sweden.

Our research group from Lund University and Region Skåne has evaluated relapse prevention as an individual treatment for problematic gaming in a randomized controlled trial among children aged 12-18 years in a child and adolescent psychiatry (CAP) setting [18]. This was one of the first treatment studies for children with problematic gaming in Europe. Although we discovered that the individual treatment of children and youth has a promising effect on their gaming, we also found that important pieces were lacking when providing treatment for children with problematic gaming. The most prominent one was the lack of family, including parents, involvement in the treatment. Indeed, children and their development need to be seen from a system perspective [19]—children develop interaction with their environment or family. For children, parents are the closest socializing agents in their environment, who, with their parenting behaviors and practices, pave the way for child developmental transitions. In that sense, the development of certain behaviors, such as gaming and excessive screen use, may, at least to some extent, be related to family factors [20,21]. It is then possible that a child’s problematic screen time or gaming is not only attributed to the child itself, but to the family as a system. Therefore, we believe that parents need to be included in the preventive interventions targeting children and their families. Instead of only focusing on the child, changing the family environment, including parent-child interactions at home, may pose significant changes in the child’s screen behavior. This is a recognized logic from other family interventions such as the Strengthening Families program targeting families with children with behavioral problems [22] and the Cool Kids program [23] targeting families with children with anxiety. In fact, parents whose children have undergone the relapse prevention treatment in our project state that they need to and want to be involved in the child’s treatment. Arguments for family-centered interventions in the treatment of IGD have been presented in previous compilations and emphasize experiences from addiction conditions, where family system involvement has been shown to be important in bringing about behavioral changes in the child [24]. Additionally, group-based treatment shows promise for adolescents with problematic gaming [25], although the underlying mechanisms need further exploration. Problematic screen use, not the least gaming, is particularly shown in children with neurodevelopmental disorders, such as attention-deficit/hyperactivity disorder and autism spectrum disorder [3,26], and we, therefore, consider it crucial to evaluate the intervention in a clinical setting where these children are present.

The objective of this study is to determine the possibility of distributing and evaluating a family-centered group program for problematic gaming and excessive screen use (FAME) in a clinical CAP population. We will examine the feasibility of the program including treatment delivery and recruitment of the participants. Additionally, we also aim to examine the participants’ experience of the program, as well as the relevance of the selected measuring instruments. The conclusions drawn from this study will serve as the foundation for a larger, quasi-experimental study in a clinical setting to follow.

## Methods

### Overview

The family-centered program for problematic gaming and excessive screen use in a child and youth population (FAME; S Kapetanovic, unpublished data, 2024) is a group-centered, family program developed and based on theoretical and empirical knowledge of parenting and child and youth development [19-21]; knowledge from the previous randomized controlled trial study on individual treatment of child and youth gaming problems [18]; and findings from interviews with the parents regarding their needs and content aspirations relating to gaming and excessive screen use.

The program specifically targets families where the child exhibits excessive screen use including problematic gaming and social media use. Both the child and at least 1 parent participate in the program, with the main goals being (1) to reduce problematic and excessive screen use in children and (2) to strengthen parent-child relationships. The logic model behind the program (Textbox 1) illustrates that these goals are achieved through various components such as psychoeducation, cognitive restructuring, and social and emotional learning. The theoretical framework is based on systemic theory [27], social cognitive theory [28], and emotion socialization theory [29]. The psychoeducational elements consist of information on screen-related health, sleep hygiene, and supportive interactions with children with mental health issues and are conveyed and illustrated through video clips, the use of a shared whiteboard, and illustrative example situations. The program uses different activities, including storytelling and on-site and home assignments, to help children and parents understand the positive and negative aspects of screen use, recognize cognitions and emotions, and prevent problems and family conflicts (proximal goals). Additionally, the program aims to teach participants how to handle emotions and behaviors as mediating mechanisms, ultimately leading to the attainment of the main or distal goals.

The program includes an initial individual session (session 0) where the child and the participating parent or parents meet with a clinician for goal setting and a motivational interview. This is followed by four group sessions with the following content: (1) positive and negative aspects of screen use and structure for setting boundaries; (2) psychoeducation on how thoughts, feelings, and behaviors influence each other and strategies for emotion regulation; (3) identifying triggers and preventing conflicts; and (4) psychoeducation on sleep hygiene and continued work moving forward.

Group session 1, conducted separately for children and parents, begins 1 to 2 weeks after session 0. Although the themes in both child and parent groups are aligned, the activities and outputs are tailored to be age appropriate and meet the specific needs of children and parents.

The sessions will take place at a CAP clinic that serves both subclinical and clinical groups of patients. The program will be delivered by clinicians, who could be doctors, psychologists, or health counselors, all possessing competence in cognitive behavioral therapy and having completed a 2-day FAME program education.

Each session is expected to last approximately 90 minutes, including a 15-minute break. This timeframe allows for a comprehensive and effective delivery of the treatment.

**Logic model of the FAME program.**

**Target group**

- Families seeking support for problematic screen use in children

**Theory**

- Family system theory
- Social cognitive theory
- Theory of emotion and emotion-focused therapy

**Components**

- Psychoeducation
- Cognitive restructuring
- Emotional knowledge and regulation
- Parenting skills

**Activities**

- Meeting sessions
- Discussions
- Storytelling
- Practice and homework

**Outputs**

- One individual meeting with the clinician
- Four group sessions with children and parents in separate groups
- Homework
- Booklets

**Proximal goals**

- Understanding the positive and negative sides of screen use
- Recognizing feelings and emotional and behavioral triggers
- Preventing emotional tantrums and family conflicts

**Mediating mechanisms**

- Children learn to control their behavior
- Parents learn to handle (child) feelings and behaviors
- Enhanced parenting skills

**Distal goals**

- Less problematic screen use
- Better parent-child relationships

### Enrollment

Primary care patients (*Första linjen*) seeking treatment at CAP units in Skåne for problematic gaming or excessive screen time will be given the opportunity to participate in our treatment program. Patients contact CAP Skåne through En Väg In, a call center managed by psychiatry nurses and health care counselors. During the standard intake interview, patients are asked “Does your child (or “do you” if the child calls themselves) have any problems related to gaming or screen use?” As part of the existing procedure, parents or children who call En Väg In are also asked if they consent to be contacted for participation in research programs. The responses are tagged by the staff, and these tags are analyzed weekly by research administrators. Families with both tags are then contacted and provided with oral and written information about the study and the program. Families can be included in the study in 2 ways: either by being contacted by the research team during the initial contact with CAP, following the procedure described above, or by their therapist at CAP. Therapists at CAP can contact the study if families have described screen-related family conflicts in the context of receiving other care and have consented to be contacted by the research team for further information about the study. Regardless of the contact method used, research team staff will contact guardians by phone in an initial call to provide information about the study, assess motivation and consent, and verify inclusion and exclusion criteria. If all inclusion criteria are met and no exclusion criteria are present, the family will be scheduled for session 0, the content of which has been described above. For this pilot study, we will enroll 10 families, involving both the child and parent or parents. The inclusion and exclusion criteria are provided in Textbox 2.

**Inclusion and exclusion criteria.**

**Inclusion criteria**

- Age between 10 and 18 years
- Reported problems in the family regarding computer gaming or screen use
- Children or youth and guardians can read, write, and communicate in Swedish
- An available guardian who can participate in the program
- Possibility to participate in program sessions on-site

**Exclusion criteria**

- Observed or suspected intellectual disability

### Measures

The following data will be collected within the framework of this pilot study. The usability of the scales will be evaluated in future analyses. The data given below will be collected from the children:

- SCREENS-Q screening form, for valuing children and adolescent’s screen time (adapted version, including questions targeting extent, type of activity, boundary setting, and positive and negative consequences of the child’s screen use): It has shown good internal consistency (Cronbach α 0.61-0.90) [30].
- Game Addiction Scale for adolescents, for the diagnosis of computer game addiction targeting questions in accordance with the proposed
  Diagnostic and Statistical Manual of Mental Disorders
  criteria for IGD [5]: The instrument has shown good internal consistency (Cronbach α 0.81-0.86) [31].
- The Kessler Psychological Distress Scale (Kessler-6) screening for depression and anxiety over the past 6 months, including questions regarding feelings of hopelessness and nervousness: It is a widely internationally used measure of psychological distress. The instrument has shown a sensitivity of 0.36 (0.08) and a specificity of 0.96 (0.02) in predicting severe mental illness in a general population [32,33].
- Parenting Children and Adolescents Scale (PARCA) for assessing aspects of the parent-child relationship and parenting practices (adapted version including questions regarding encouraging positive behaviors and setting boundaries) [34]: This is originally a 19-item measure with each item rated on a 5-point scale. The instrument includes 3 subscales: encouragement of positive behaviors, setting limits, and proactive parenting behaviors, and has shown good psychometric properties (Cronbach α 0.86-0.91) [34].
- Adult-Child Relationship Scale—Family Check-Up (FCU): It measures warmth and conflict with 8 items rated on a 5-point scale targeting questions such as the tendency to involve parents in the child’s thoughts and feelings and 3 items measure parent-child conflicts and conflict behaviors. Scale α values in previous studies have shown good internal consistency (Cronbach α 0.74-0.85) [35].
- The Insomnia Severity Index Adolescent for assessing sleep problems: It is a well-established tool for measuring sleep problems in adolescents, including questions targeting the child’s perception of the consequences of their sleep patterns. Scale α values in previous studies have shown good internal consistency (Cronbach α 0.83) [36,37].
- Background data: gender, age, length, weight, family constellation, parental education level, and diagnosis at CAP.

Baseline data that will be collected from the parents before treatment are:

- SCREENS-Q screening form for valuing children and adolescents’ screen time (adapted version, including questions targeting extent, type of activity, boundary setting, and positive and negative consequences of the child’s screen use) [30].
- PARCA for assessing aspects of the parent-child relationship and parenting practices (adapted version including questions regarding encouraging positive behaviors and setting boundaries) [34].
- FCU: 5 items measure parent-child warmth such as questions about the parent’s perception of the child’s openness about their thoughts and feelings, and 3 items measure parent-child conflicts and conflict behaviors [35].

Given our expectation that a larger evaluation will be able to determine whether the intervention has any impact on screen-related conflicts within families, we will pay particular attention to the usability of the PARCA and FCU instruments. The follow-up data will be collected in connection with the last session of the program. Interviews with children and parents will be conducted approximately 2 weeks after the end of the program. The interviews will focus on the participant’s experience of the program. The quantitative data will be analyzed with SPSS (IBM Corp), and the interview data will be analyzed with thematic analysis [38].

### Analysis

In our analysis, we will be addressing several key indicators such as (1) monitoring the recruitment rate by tracking the number of eligible participants approached versus the number enrolled in the study; (2) tracking the retention and attendance rates of both parents and children; (3) evaluating whether each session’s objectives are met, the content is delivered within the allotted time, and the necessary resources (eg, facilitators and materials) are available; and (4) gathering qualitative and quantitative feedback from participants through postprogram surveys and individual interviews with both children and parents. By assessing these criteria, we will determine if the program and recruitment processes are feasible and ready for expansion into a larger, quasi-experimental study. In order to capture the participants’ experience of the program, semistructured interviews will be conducted and analyzed using thematic analysis [38]. For the statistical analysis, we will use 2-tailed paired sample *t* tests to address potential differences in pre- and postprogram measures. However, if data are not normally distributed, a nonparametric Wilcoxon signed rank test will be used. After each session, group leaders together with the project owner will assess compliance and pay particular attention to any adverse events such as deterioration in family relationships or increasing ill health of the participants. What constitutes an adverse event in psychotherapy can be difficult to define [39], although it could constitute severe illness, parental divorce, etc. We will, therefore, pay particular attention to any of the changes within the individuals and their families in the various parts of the data collection.

### Ethical Considerations

The study was reviewed and approved by the Swedish Ethical Review Authority (2023-05533-01; November 13, 2023). The study has been registered at ClinicalTrials.gov (NCT06098807) and approved on October 23, 2023. We have calculated that the risk for patients to participate in the study is low. Mental illness among young people causes great distress and costs both on individual and societal levels. The knowledge and understanding of mental illness among young people are low in general society and particularly low regarding screen behaviors among youth. Hence, new knowledge about treatment of excessive screen use is of uttermost importance and the benefits will be greater than the risks of this project. The trial intervention is similar to other clinical practices offered in child and youth psychiatric clinics, which is why we consider the risks with the trial as minimal. Participants will not receive any economic compensation for their participation in the study. After eligibility is confirmed, written and verbal information about the study will be provided to all participants according to the Swedish Act concerning the Ethical Review of Research Involving Humans (SFS 2003:460). All patients and their carers who verbally agree to take part in the project will be provided with a consent form enabling them to provide written consent. According to Swedish law on research ethics, for patients younger than 15 years of age, written consent from a parent or guardian is also required for their children to participate in the study. All participants will be informed that their partaking in the study is voluntary; their data will be handled with strict confidentiality; results will be reported solely on a group level, which means that individual participants will not be identifiable; and that they are free to withdraw from the study at any time without reporting a reason for withdrawal. Personal and identifiable data will be collected from patients. Data will be kept confidential and managed in accordance with the Data Protection Act, General Data Protection Regulation policies, and the Swedish Act concerning the Ethical Review of Research Involving Humans (SFS 2003:460). Data will be held on services located within the Region Skåne databases, stored and secured both physically (in locked cabinets designed for the purpose) and electronically (behind firewalls), and be accessible to the research team only.

## Results

A total of 11 children and their parents were enrolled during the first quarter of 2024. A 4-session pilot was delivered in the first quarter 2024, and the first results are expected in the third quarter 2024. The results are intended for publication in international peer-reviewed scientific journals and presentation at international and national scientific conferences. A primary publication is planned for the first quater of 2025. Additional secondary publications may follow. Whenever possible, the results will be presented in open-access journals.

## Discussion

### Expected Outcomes

This study aims to evaluate the delivery and feasibility of the newly developed family-centered group program (FAME) designed to address problematic gaming and excessive screen use among children and adolescents aged 10-18 years within a CAP setting. The program is based on established empirical knowledge in the field (Textbox 1) and our own experiences from previously completed studies [18,40,41]. We anticipate that (1) the program will demonstrate feasibility in terms of treatment delivery and participant recruitment, (2) participants’ experiences with the program will provide valuable insights for further development and testing, and (3) the selected measurement instruments will be relevant and effective in evaluating program outcomes.

Our intention is that both the quantitative and the qualitative exploration of the pilot study will form an important basis for refining the program, as well as for the development of a larger study. Based on the low number of participants, it will not be possible for us to draw any conclusions regarding the program’s effect. However, we expect to observe a decrease in children’s problematic screen use, including a reduction in the amount of time spent using screens. Additionally, we anticipate improvements in the parent-child relationship, characterized by reduced conflict and increased family cohesion. If these criteria are met, we will consider modifying the program as needed and proceed to a fully powered study.

Despite the huge interest in children and adolescents’ screen behavior in both popular science and more clinical and scientific contexts, to date, there is neither consensus regarding what is acceptable screen time nor evaluated family interventions for problem gaming and excessive screen use. Child screen use is generally seen as both a cause and a consequence of problematic parent-child relationships [42,43], and family interventions, where both the child and the parents are included, are both recommended [24] and requested [40]. Therefore, this would be the first intervention of a kind that could be of help to both children and their parents. Such an intervention could be beneficial for individual families, but it could also be an effort that would contribute to reduced health inequalities among children and families.

### Limitations

One limitation is the small number of families included in the pilot study. Notably, this pilot study is primarily exploratory in nature and our focus is on assessing the format and practical implementation of the intervention rather than drawing conclusions about its efficacy. Another potential challenge that arises involves differing perceptions between parents and children concerning the child’s health and the dynamics of the parent-child relationship [44]. To address this issue, we approach it by soliciting perspectives from both the child and parent regarding screen use and their parent-child relationship, without biasing our interpretation toward either viewpoint. Additionally, based on our observations and past clinical encounters, we anticipate potential differences in motivation between parents and children, with parents often showing greater enthusiasm for participation in our program than their children. Our strategy involves proactively addressing and managing such scenarios during the initial individual session with the therapist prior to commencing group sessions.

### Conclusions

This unique intervention involves both the child and at least 1 parent, with the multiple aims of reducing problematic screen use, enhancing parent-child interactions, and mitigating conflicts related to screen use. The findings from this study will inform subsequent research efforts, which will aim to conduct a larger-scale evaluation of the intervention’s effectiveness in reducing family conflicts associated with screen time. Such an early intervention targeting both children and their parents has the potential to be an important support for many families and be relevant to several of society’s support functions.

## Abbreviations

CAP: child and adolescent psychiatry
FCU: Family Check-Up
IGD: internet gaming disorder
PARCA: Parenting Children and Adolescents Scale

## Data Availability

The datasets generated during or analyzed during this study are available from the corresponding author upon reasonable request.
